# The crosstalk between acute hepatopancreatic necrosis disease (AHPND) and the *Vibrio* quorum sensing (QS) system: A review

**DOI:** 10.1371/journal.ppat.1013980

**Published:** 2026-02-20

**Authors:** Hao-Ching Wang, Ramya Kumar, Michael Eniola Ayenero, Kai-Cheng Hsu, Chu-Fang Lo, Shin-Jen Lin, Han-Ching Wang

**Affiliations:** 1 Graduate Institute of Translational Medicine, College of Medical Science and Technology, Taipei Medical University, Taipei, Taiwan, ROC; 2 The PhD Program for Translational Medicine, College of Medical Science and Technology, Taipei Medical University and Academia Sinica, Taipei, Taiwan, ROC; 3 International PhD for Translational Science, College of Medical Science and Technology, Taipei Medical University, Taipei, Taiwan, ROC; 4 International Center for the Scientific Development of Shrimp Aquaculture, National Cheng Kung University, Tainan, Taiwan, ROC; 5 Department of Biotechnology and Bioindustry Sciences, College of Bioscience and Biotechnology, National Cheng Kung University, Tainan, Taiwan, ROC; 6 Graduate Institute of Cancer Biology and Drug Discovery, College of Medical Science and Technology, Taipei Medical University, Taipei, Taiwan, ROC; 7 Ph.D. Program for Cancer Molecular Biology and Drug Discovery, College of Medical Science and Technology, Taipei Medical University, Taipei, Taiwan, ROC; 8 Biomedical Commercialization Center, Taipei Medical University, Taipei, Taiwan, ROC; Monash University, UNITED STATES OF AMERICA

## Abstract

Acute hepatopancreatic necrosis disease (AHPND) is a disease that has caused significant losses to shrimp farming since 2009. The primary mechanism of this disease involves the binary toxins PirA^*vp*^ and PirB^*vp*^, which are produced by specific strains of *Vibrio parahaemolyticus*, and which lead to significant damage to the hepatopancreatic cells of shrimps. Recent studies on the pathology of AHPND have also highlighted the role of the *Vibrio* quorum sensing (QS) system, which affects growth, virulence, and biofilm regulation in *Vibrio* species. For example, deletion of the *qseC* gene reduces the virulence of the AHPND-causative *V. parahaemolyticus*. Most importantly, the QS regulators LuxO^*vp*^ and AphB^*vp*^ have been implicated as they control the growth-phase-dependent expression of the *pirA*^*vp*^*/pirB*^*vp*^ genes. Additionally, given the growing problem of antibiotic resistance, this article reviews several alternative control strategies targeting the QS system, including QS inhibition using natural products, biofloc technology, and the development of small-molecule inhibitors against AphB^*vp*^. Finally, we also discussed the potential of using probiotics to enhance shrimp disease resistance through QS inhibition, highlighting the feasibility of targeting the QS system for AHPND control.

## Introduction

Over the last decade, there has been significant growth in the shrimp industry, with the Southeast Asian countries continuing to be among the major producers of shrimp, contributing about 840,000 metric tonnes to the global output in 2023 [1]. Although there is an overall trend of increasing shrimp production, factors such as feed costs and the broodstock quality remain top concerns for the industry. Moreover, both newly emerging and existing diseases pose an even greater impact on shrimp production [1]. Acute hepatopancreatic necrosis disease (AHPND) in particular has been responsible for significant global losses in the shrimp farming industry since 2009 [2–5]. The primary symptom of AHPND is damage to the hepatopancreatic cells of the infected shrimp [6,7], causing the compromised hepatopancreatic tissue to subsequently lose its digestive and immune functions. The disease affects the two major farmed shrimp species, *Litopenaeus vannamei* and *Penaeus monodon,* and leads to outbreaks with mortality rates ranging from 70% to 100% [2–5]. Recent reports suggested that between 2009 and 2018, losses of US$4 billion/year were attributable to white spot disease (WSD) and AHPND [8,9]. AHPND alone, which specifically targets early-stage shrimp, causes production losses that are now estimated at around 1 billion US dollars annually [9]. The biological severity of this disease has translated directly into operational devastation, wiping out entire production cycles. Consequently, AHPND is widely regarded as one of the most formidable threats to shrimp aquaculture, not only due to financial loss but also through secondary ecological and social-economic ramifications that disrupt local aquaculture practices and destabilize the livelihood of communities dependent on shrimp farming.

AHPND is caused by specific pathogenic strains of *Vibrio parahaemolyticus* [6,8,10]. *V. parahaemolyticus* is a halophilic, Gram-negative bacterium that naturally occurs in seawater. Initially, certain strains were identified as opportunistic pathogens responsible for human gastroenteritis and other symptoms caused by consuming contaminated seafood [11], but after the appearance of AHPND, other specific strains of *V. parahaemolyticus* were also identified as the cause of this serious shrimp disease. The AHPND-causing strains of *V. parahaemolyticus* were found to contain a ~70 kbp plasmid called pVA1 [4,6,10]. Among the 40 functional genes on pVA1, two Photorhabdus insect-related (Pir) binary toxins (PirA^*vp*^ and PirB^*vp*^) were identified as the primary cause of AHPND symptoms, specifically the disease-induced damage to the cells of the hepatopancreas [4–6,12]. It was also found that pVA1-like plasmids could be passed among different *Vibrio* species through horizontal transfer [13–16], and that any *Vibrio* species capable of expressing the complete *pirA*^*vp*^ and *pirB*^*vp*^ genes could cause hepatopancreatic necrosis [6,17–20]*.* Structurally, PirA^*vp*^ and PirB^*vp*^ toxins are similar to *Bacillus thuringiensis* Cry pore-forming toxins, suggesting they act by forming pores in cell membranes. This mechanism involves complex formation, receptor binding, and subsequent oligomerization to create uncontrolled pores leading to cell death [5,6]*.* Given their importance for AHPND, the cytotoxic and regulatory mechanisms of the *pirA*^*vp*^ and *pirB*^*vp*^ genes have subsequently attracted much interest, and recent studies have shown how quorum sensing (QS) systems play an important role in AHPND pathogenesis [21–27]. In this review article, we will focus on the interactions between AHPND and the *Vibrio* QS system.

## Quorum sensing (QS) in *Vibrio* spp

**Fig 1:**
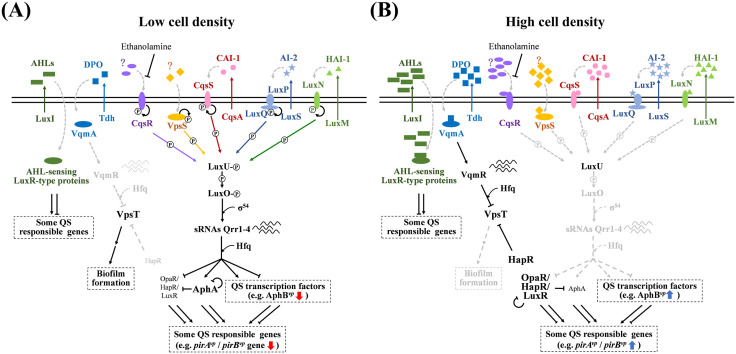
Schematic representation of the quorum sensing system for *Vibrio* spp. under low cell density (A) and high cell density (B) conditions. In *V. cholerae*, five sensors/receptors (LuxP/LuxQ, CqsS, VpsS, CqsR, and VqmA) have been identified. Among them, LuxP/LuxQ, CqsS and VqmA can recognize the autoinducers (AIs) AI-2, CAI-1 and DPO, respectively [which are synthesized by LuxS, CqsA and threonine dehydrogenase (Tdh), respectively]. However, as indicated by question marks, the AI synthases and the AIs for VpsS and CqsR remain unknown. In *V. parahaemolyticus* and *V. harveyi*, the presence of LuxN, LuxP/LuxQ, and CqsS can recognize HAI-1 (an AHL synthesized by LuxM), AI-2, and CAI-1. Additionally, in *V. fischeri*, besides AI-2, the AHLs synthesized by LuxI can be recognized by AHL-sensing LuxR-type proteins. **(A)** Under low cell density conditions, the AIs are absent or at low concentrations, resulting in the autophosphorylation of LuxN, LuxQ, CqsS, VpsS and CqsR. The phosphate group is transferred by a phosphotransfer protein, LuxU, to the core regulator of the quorum sensing system, LuxO. The phosphorylated LuxO (LuxO~P) then acts in combination with the alternative sigma factor σ54 to drive the expressions of the small RNAs (sRNAs) Qrr 1-4. The sRNAs interact with an RNA chaperone, Hfq, to stabilize AphA mRNA, and promote degradation of OpaR/HapR/LuxR (which belong to the TetR family, different from the AHL-sensing LuxR-type proteins [53]) mRNA, as well as regulate other QS transcription factors. These transcription factors further regulate hundreds of genes that are involved in many physiological processes. Meanwhile, VqmA remains in a state of low activity without DPO binding, and the expression of another sRNA, VqmR, is suppressed. This results in the expression of VpsT, which is an important transcription activator of biofilm formation. Similarly, without binding to AHL, AHL-sensing LuxR-type proteins also remain in an inactive conformation, affecting the expression of downstream genes. **(B)** Under high cell density conditions, in contrast, the sensors/receptors convert to phosphatases, which results in the dephosphorylation of LuxU and LuxO. Since the Qrrs are no longer expressed, the stability of AphA mRNA is decreased, and the suppression of OpaR/HapR/LuxR mRNA is canceled. In addition, the accumulated DPO binds to VqmA, which triggers the expression of VqmR. The VqmR in turn binds with Hfq to suppress the expression of VpsT. The presence of HapR also suppress the expression of VpsT. Moreover, the accumulated AHLs can diffuse into cells, bind to the AHL-sensing LuxR-type proteins, and subsequently regulate the transcription of the downstream genes.

The QS system is essential for microbial communication, allowing bacteria to coordinate actions based on the concentration of diffusible signal molecules in their environment [28]. The system regulates a diverse array of cellular activities in both Gram-positive and Gram-negative bacteria, including virulence, symbiosis, antibiotic resistance, and biofilm formation [28]. QS has been the focus of extensive research in key bacterial species such as *Staphylococcus aureus*, *Pseudomonas aeruginosa*, *V. harveyi*, and *V. cholerae* [29,30]. In *Vibrio*, the classic multi-channel QS system is regulated by factors such as LuxN, LuxPQ, CqsS, LuxU, LuxO, as well as the master transcriptional regulators known as AphA and LuxR. LuxR here is a TetR family protein with different names in different species: it is known as LuxR in *V. harveyi* and *V. alginolyticus*, OpaR in *V. parahaemolyticus*, HapR in *V. cholerae*, and SmcR in *V. vulnificus* [31]. As noted above, the QS system also relies on diffusible signal molecules known as autoinducers (AIs). Bacteria synthesize and release these AIs, and since they can also be detected and responded to by neighboring cells within a mixed microbial community, this facilitates intercellular communication between adjacent cells. When cell density is low, AI concentration decreases, which leads to activation of kinase activity in the transmembrane receptors LuxN, LuxPQ, and CqsS. These receptors first undergo autophosphorylation and then phosphorylate LuxO via LuxU. Phosphorylated LuxO activates quorum-regulatory sRNAs (Qrr sRNAs) which affect the stability of LuxR mRNA, thereby reducing the expression of LuxR protein and increasing the expression of AphA (Fig 1A). Conversely, at high cell density, the accumulated AIs facilitate the binding of HAI-1 [a kind of N-acyl-homoserine lactone (AHL), synthesized by LuxM], AI-2 (synthesized by LuxS), and CAI-1 (synthesized by CqsA) to the LuxN, LuxPQ, and CqsS receptors, respectively, causing these receptors to switch from kinase to phosphatase activity. This further leads to the dephosphorylation and inactivation of LuxO. Under these conditions, the Qrr sRNAs are not produced, AphA is no longer activated, and the translation of LuxR is no longer repressed [32–34] (Fig 1B). In addition to HAI-1, AI-2, and CAI-1 mentioned above, other AIs may be generated in *Vibrio*. For example, *V. cholerae* has also been reported to generate DPO (3,5-dimethylpyrazin-2-ol) [35] and unknown AI molecule(s) that can be detected by two membrane protein receptors, CqsR and VpsS [36]. As the earliest described species possessing a QS system, *V. fischeri* produces AHLs [37]. Moreover, Muthukrishnan and colleagues has reported that all the AHPND positive strains they examined (*V. parahaemolyticus* and *V. harveyi*) were able to produce AHL [23]. To better understand this complex network, we summarized the above information in Fig 1.

AphA and LuxR are the master transcription factors in the QS system, controlling the expression of hundreds of genes [33,38–40], including many virulence genes in *Vibrio* species such as the genes in type-III secretion systems (T3SS1 and T3SS2), type-VI secretion systems (T6SS1 and T6SS2), and the thermostable direct hemolysin genes (*tdh1* and *tdh2*) [32,34,41,42]. Beyond the secretion systems and hemolysins, the QS regulon in pathogenic *Vibrio* species also encompasses a wide array of virulence determinants. These include extracellular proteases such as hemagglutinin/protease (HapA) and metalloproteases, which contribute to tissue damage and nutrient acquisition [32,34,38,39]. The QS further regulates the production of siderophores, which are essential for iron scavenging in an iron-limited host environment [39,43], as well as other genes encoding pili and other adhesins that facilitate host cell attachment [26,39]. Multiple factors involved in immune evasion are also controlled by the QS, highlighting its broad regulatory scope in *Vibrio* pathogenesis. Meanwhile, it is of particular interest to this review that the pathogenicity of *Vibrio* spp. is affected by biofilm formation, which is also under the control of the QS system [38,44]. Biofilm formation is a rather complex cellular process, and since different bacteria have different growth or infection patterns, the regulation of biofilm formation also varies [45]. For example, at low cell density, the absence of QS autoinducers activates AphA and suppresses OpaR, which further inhibits biofilm formation in *V. parahaemolyticus* [46]. Also, the inhibition of LuxR in *V. harveyi* acts to suppress biofilm formation [47]. In contrast, a high level of AphA combined with a low level of HapR is associated with increased biofilm formation in *V. cholerae* [48].

At high cell density, OpaR has been shown not only to increase the level of cellular cyclic di-GMP (c-di-GMP), a key secondary signaling molecule for biofilm formation [38], but also to inhibit swarming motility by directly repressing lateral flagellar (Laf) genes [49] and to negatively regulate swimming motility by repressing polar flagellum (Pof) genes [50]. These mechanisms upregulate biofilm formation in *V. parahaemolyticus* [46]. Conversely, in *V. cholerae* at high cell density, HapR induces the expression of motility-related genes in (such as genes responsible for flagella production) [51], while inhibiting biofilm formation, all of which promotes bacterial dispersal [36,52].

Such differences presumably arise because different bacteria have different survival/infection strategies. Bacteria like *V. parahaemolyticus* and *V. harveyi* only initiate biofilm formation after finding a suitable environment and proliferating, thereby protecting the large number of cells within the biofilm matrix. This approach avoids wasting energy on biofilm formation during resource-scarce conditions, and thus delivers a collective advantage. Conversely, for pathogens like *V. cholerae*, the biofilm formed at low cell density protects the bacteria from external environmental stresses (including those in bodies of natural water and those in the internal environment of the host) and facilitates both their survival and colonization. Subsequently, once bacterial numbers increase, bacterial dispersal becomes necessary, so as cell density increases, biofilm formation is reduced [53]. This comparative analysis shows how the QS-regulated lifestyle of AHPND-causing *V. parahaemolyticus* is characterized by enhanced biofilm formation at high population density, and how this specific strategy contributes to virulence and disease development in shrimp aquaculture.

## The QS system and AHPND pathogenesis

**Fig 2:**
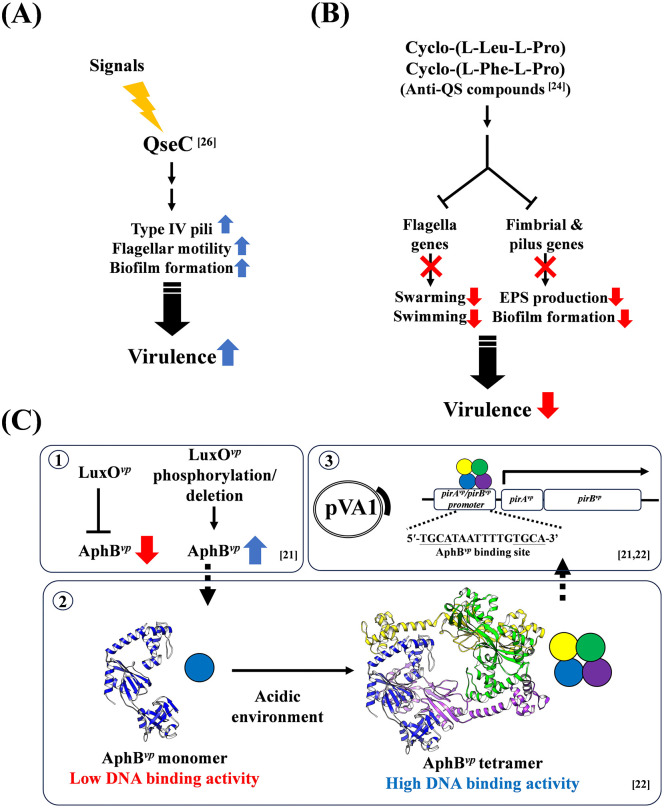
Our current understanding of how the QS system is involved in AHPND pathogenesis. **(A)** A sensor kinase, QseC, positively regulates the genes involved in type IV pili, flagellar motility and biofilm formation, which may further upregulate the virulence of AHPND-causing *Vibrio parahaemolyticus*. The input signals to QseC are the host catecholamine stress hormones (epinephrine, norepinephrine, dopamine) and autoinducer-3 (AI-3). **(B)** The anti-QS compounds identified from *V. alginolyticus* BC25 extract, Cyclo-(L-Leu-L-Pro) and Cyclo-(L-Phe-L-Pro), also inhibit several physical processes and downregulate the virulence of AHPND-causing *V. parahaemolyticus*. The inhibited physical processes include motility (swarming and swimming) and biofilm formation, while there is also a decrease in the production of extracellular polymeric substances (EPS), which are organic polymers of microbial origin involved in bacterial interactions with the environment. **(C)** The new understanding of *pirA*^*vp*^/*pirB*^*vp*^ gene regulation: (1) Since LuxO^*vp*^ is a negative regulator of AphB^*vp*^, its removal or phosphorylation induces an increase in AphB^*vp*^ expression. (2) The low DNA binding ability of monomeric AphB^*vp*^ improves when it forms a tetramer, and an acidic environment induces the formation of AphB^*vp*^ tetramers. All spheres correspond to AphB^*vp*^, which forms a homotetramer. The different colors represent each individual AphB^*vp*^ monomer within the tetrameric assembly. (3) Tetrameric AphB^*vp*^ binds to a specific DNA sequence on the *pirA*^*vp*^*/pirB*^*vp*^ promoter and promotes the expression of the toxin genes.

The significance of the QS system in AHPND pathogenicity was first suggested by Yang and colleagues [26] in a study which found that the deletion of the gene for *qseC* (which is considered to be a sensor kinase in the QS system [54]) reduced the virulence of AHPND-causing *V. parahaemolyticus.* The QseC protein acts as a crucial bacterial receptor that senses both host catecholamine stress hormones (epinephrine, norepinephrine, and dopamine) and bacterial autoinducer-3, thereby influencing bacterial virulence by regulating processes like growth and motility. It also positively regulates several virulence genes involved in type IV pili, flagellar motility, and biofilm formation [26] (Fig 2A). A subsequent study reported that an extraction of *V. alginolyticus* BC25 containing anti-QS compounds inhibited the virulence of AHPND-causing *V. parahaemolyticus* [24]. Transcriptomic analysis further revealed that the *V. alginolyticus* BC25 extract significantly reduced the expression of flagella genes involved in biofilm formation as well as other virulence genes found in AHPND-causing *V. parahaemolyticus* [24] (Fig 2B)*.* In 2022, Muthukrishnan and colleagues reported increased expression of the QS master regulatory gene *luxR* and the transmembrane transcriptional regulatory gene *toxR* during infection with AHPND-causing *V. parahaemolyticus* and *V. harveyi* [23]. However, while these studies suggested a link between the QS system and the pathogenicity of AHPND-causing *V. parahaemolyticus*, direct evidence was still lacking.

More recently, our studies have provided further details of how the QS system is involved in regulating AHPND PirA^*vp*^/PirB^*vp*^ expression [21,22]. As detailed in our previous study by Lin and colleagues [21], the expression levels of *pirA*^*vp*^ and *pirB*^*vp*^ genes were low during the lag phase (between 0-3.5 hours). The expression levels of these genes then peaked and remained high during the early log phase (between 3.5 and 6 hours) the highest levels of mRNA and proteins were detected at this phase. Subsequently, as growth progressed and the cell density increased (between 6 and 13 hours), the expression of these toxins was significantly repressed, resulting in low transcript levels at the stationary stage. We then verified that at early log phase, the deletion of LuxO^*vp*^ significantly increased the gene and protein expression of PirA^*vp*^ and PirB^*vp*^ in *V. parahaemolyticus.* This suggests that LuxO^*vp*^ is a negative regulator of the toxin genes and explains why these genes are expressed during the early log phase but repressed at high cell density. Next, the QS regulator AphB^*vp*^ (*V. parahaemolyticus* AphB transcriptional factor) was shown to bind with the 5′-TGCATAATTTTGTGCA-3′ sequence in the promoter region of the *pirA*^*vp*^/*pirB*^*vp*^ genes [2 1]. LuxO^*vp*^ deletion also increased AphB^*vp*^ expression, indicating that LuxO^*vp*^ negatively regulates this gene. In a subsequent study, we further confirmed that AphB^*vp*^ enhances *pirA*^*vp*^/*pirB*^*vp*^ gene expression [22].

AphB-type transcription factors belong to the LysR-type transcriptional regulator family (LTTR) [55]. LTTRs are abundant transcriptional factors in prokaryotes, and they regulate diverse genes involved in virulence, metabolism, the QS system, and motility [56–58]. This type of transcriptional factor comprises approximately 330 amino acids and typically has two conserved domains: an N-terminal winged-helix-turn-helix (wHTH) DNA binding domain, and a C-terminal effector binding domain that is involved in ligand recognition and the regulation of DNA binding domain activity [56–58]. LTTRs usually bind with DNA via their tetrameric conformation, and the binding patterns are affected by co-inducers that influence the binding of RNA polymerase and the initiation of transcription [56,58].

Compared to other LTTRs, AphB has been studied less extensively. Nonetheless, AphBs are known to usually serve as gene enhancers. In *V. cholerae*, AphB activates the *tcpPH* operon on the VPI (*Vibrio* pathogenicity island), initiating the virulence cascade in cooperation with the QS transcriptional activator AphA [59–61]. AphB was also found to enhance virulence of *V. cholerae* under anaerobic conditions [62]. The same study further revealed that the C235 residue of *V. cholerae* AphB plays an important role in sensing oxygen and modulating activity, and that the AphB C235S mutant activated virulence genes even in aerobic conditions [62]. In *V. alginolyticus*, AphB is essential for LuxR and exotoxin Asp expression [63]. Additionally, AphB contributes to the survival of *V. vulnificus* and *V. cholerae* in acidic environments [64]. For example, when faced with acidic stress, AphB activates another transcription factor, CadC, to promote the expression of CadA (lysine decarboxylase) and CadB (cadaverine/lysine antiporter), thereby increasing the pH value around the bacteria and enhancing their ability to survive in the acidic environment [65,66].

In our 2024 report [22], we confirmed that the binding between AphB^*vp*^ and its target DNA activated the expression of downstream genes. Additional results showed that AphB^*vp*^ deletion rendered the AHPND-causing *V. parahaemolyticus* unable to express the PirA^*vp*^/PirB^*vp*^ toxin genes/proteins. We further noted that the expression of these two toxin genes/proteins was increased at low pH, and to investigate this pH-dependent regulation, we determined the structure of AphB^*vp*^ using Cryo EM. We found that, like other AphBs in *V. vulnificus* and *V. cholerae*, AphB^*vp*^ has a tetrameric conformation [22]. Our results further showed that this tetrameric conformation, which is important for DNA binding activity in the LTTR family, was more likely to form as the environmental pH became lower. This was consistent with our DNA binding assay, which showed that the binding between AphB^*vp*^ and DNA was significantly increased at low pH. Our current understanding of the purported transcriptional regulation of PirA^*vp*^/PirB^*vp*^ by the QS system is summarized in Fig 2C.

The regulatory influence of pH on AphB^*vp*^ also highlights a critical intersection between aquaculture pond conditions and QS-regulated virulence. Other environmental factors such as temperature and salinity have also been shown to influence the growth and pathogenesis of *V. parahaemolyticus* and related *Vibrio* species [67–69]. As halophilic bacteria, *Vibrio* species are highly sensitive to these parameters, which modulate both their survival in marine environments and their virulence in aquaculture systems [67,69]. For instance, while *V. parahaemolyticus* demonstrates robust growth across various conditions, higher temperatures significantly accelerate development, with the stationary phase being reached faster at 35 °C than it is between 25 and 30 °C [70]. Similarly, increased salinity also enhances growth rates, with 6% NaCl yielding faster growth than lower concentrations [67]. It is important to note that the expression of the *pirA*^*vp*^ and *pirB*^*vp*^ genes is affected by temperature [70,71]. In addition, salinity has been shown to influence the expression of the *pirA*^*vp*^ gene [70]. These findings suggest that climate-driven shifts in coastal salinity and temperature are likely to increase the global risk of vibriosis outbreaks as well as enhance AHPND pathogenesis.

## The QS system could be a target for AHPND control

In recent years, due to the increasing incidence of antimicrobial resistance, there has been a pressing need to develop alternative strategies to control aquatic animal diseases. QS inhibition is envisioned as a new target for developing effective therapies against infection [72,73]. Disruption of the QS system by inhibiting AI production, degrading AI molecules, and blocking the binding of AI molecules to receptors will all reduce the virulence of the pathogenic bacteria, making them more susceptible to the host immune response and easier to clear.

Natural products from plants are particularly promising candidates for QS inhibition as they already possess anti-inflammatory, antioxidant, and anti-bacterial properties [74], and several plant-based therapeutics have already been shown to disrupt AHPND pathogenesis via QS inhibition. For instance, indole, which serves as an *Escherichia coli* QS inhibitor by disrupting the folding of the QS regulator AqsR [75], was shown to reduce the virulence of AHPND-causing *V. parahaemolyticus* [25]. An indole derivative, indole-3-acetic acid (IAA), reduces swimming motility and biofilm formation in *Vibrio* spp., while in AHPND-causing *V. parahaemolyticus*, treatment with IAA reduced mRNA levels of the *pirA*^*vp*^ and *pirB*^*vp*^ toxin genes to 46% and 42%, respectively [27].

Numerous other studies have also identified potent QS inhibitors that target *Vibrio* species, particularly those disrupting the AI-2 signaling that is central to virulence in *V. parahaemolyticus*, *V. harveyi*, and related pathogens. For example, cinnamaldehyde and its analogs, such as 2-nitro-cinnamaldehyde and 4-nitro-cinnamaldehyde, have been demonstrated to effectively inhibit QS by covalently modifying LuxR via Michael addition to cysteine residues, thereby reducing DNA-binding activity without impacting bacterial growth or autoinducer production [76,77]. These compounds suppress key virulence traits such as protease and pigment production in *V. anguillarum* as well as biofilm formation in both *V. vulnificus* and *V. anguillarum,* and they heighten *Vibrio* susceptibility to oxidative stress. Importantly, they have been shown to protect model hosts like *Artemia nauplii* and *Caenorhabditis elegans* from lethal *Vibrio* infections *in vivo* [76,77]. Defoirdt and colleagues, also demonstrated that brominated thiophenone compounds (e.g., TF310 and TF101) exhibited broad-spectrum QS antagonism across HAI-1, AI-2, and CAI-1 signal systems in *V. harveyi* at low micromolar concentrations (IC50 ~2.5 μM), achieving complete protection of brine shrimp against luminescent vibriosis with a therapeutic index exceeding 100 and negligible host toxicity [78,79]. Structure-activity relationships have further underscored the role of α,β-unsaturated carbonyls and electron-withdrawing groups in enhancing LuxR affinity [31,80] while synergizing with indoles and bioflocs for non-bactericidal anti-virulence therapies against *Vibrio* biofilms and pathogenesis. These and other inhibitors, including natural furanone derivatives (5Z)-4-bromo-5-(bromomethylene)-3-butyl-2(5H)-furanone, QStatin, and quoromycin, minimize resistance risks in *Vibrio* species and offer promising alternatives to antibiotics [79,81,82].

Another promising strategy to inhibit the QS system is through the use of biofloc technology [83]. This technology provides aquaculture with a sustainable method to recycle waste nutrients, particularly nitrogen from uneaten feed and shrimp excreta, into a microbial biomass with a balanced carbon-nitrogen ratio [84,85]. The system is typically maintained by adding a source of carbon (e.g., glucose) to create a dense heterotrophic community of bacteria, algae, protozoa, and organic particles known as biofloc. The bacteria community assimilates ammonia into biomass via carbon, while nitrifiers convert ammonia to nitrates and maintain key parameters such as pH (between 6.8 and 8.0), temperature (between 28 and 30 °C), dissolved oxygen (>4 mg/l), and total solids (<500 mg/l) [84]. The flocs also enable minimal or low water exchange in high-density shrimp aquaculture, which enhances biosecurity by limiting pathogen entry, boosts shrimp growth, and maintains feed efficiency [86]. Studies have found that biofloc treatment improved the survival rate of shrimp exposed to *V. parahaemolyticus*, and this was thought to be due to lowered expression of the PirB^*vp*^ toxin gene and the virulence factor genes T6SS1 and T6SS2 as a result of reduced QS regulatory gene activity [83,87]. These findings further support the idea that an AHPND control strategy that targets the QS system is feasible.

AphB^*vp*^, which directly controls the expression of the *pirA*^*vp*^ and *pirB*^*vp*^ genes, should also be a good target for small-molecule inhibitors against AHPND. Although relevant AHPND research is still lacking, AphB-type transcription factors are already considered as potential targets for anti-*Vibrio* drug development in humans [88]. For instance, the FDA-approved antiviral medication ribavirin has been shown to inhibit the production of cholera toxin by antagonizing AphB in *V. cholerae* [88]. Ribavirin does not directly affect the DNA-binding ability of *V. cholerae* AphB (AphB^*vc*^) but acts instead by binding to the C-terminal effector binding domain of AphB^*vc*^ that controls polymer formation and conformational changes. Another potential inhibitor of AphB^*vc*^ is resveratrol (3,5,4′-trihydroxy-trans-stilbene). This phytochemical is widely present in the skin of grapes and berries, and its relatively low cost would make it an attractive therapeutic for aquacultural applications. Resveratrol has various pharmacological properties, including anti-inflammatory, antiviral, antioxidant, and antimicrobial effects, and it has also been shown to inhibit biofilm formation in *V. cholerae* [89]. Molecular docking analysis identified AphB^*vc*^ as resveratrol’s possible target, as it showed the lowest binding free energy (−8.0 kcal/mol) and formed hydrogen bonds with specific amino acids in the DNA binding domain of AphB^*vc*^, which is already known to affect the regulation of *V. cholerae*’s pathogenicity [89].

**Fig 3:**
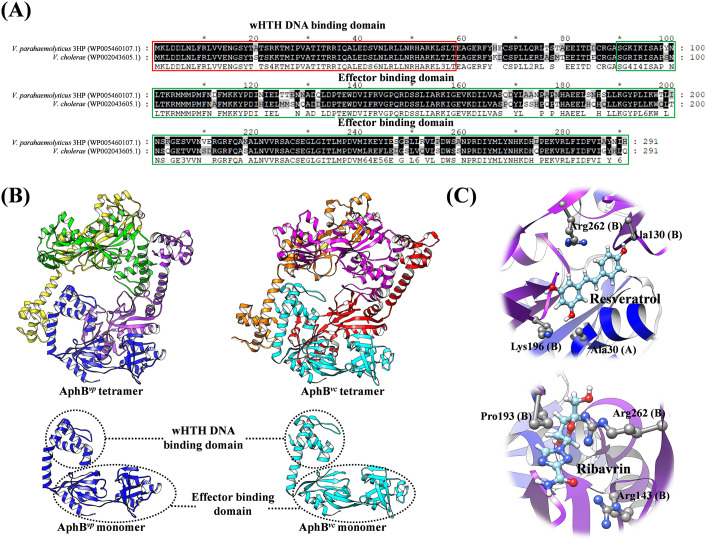
Drug development concepts based on the structure of AphB^*vp*^. **(A)** A sequence alignment between AphB^*vp*^ and AphB^*vc*^. The sequence identity and similarity are 81% and 90%, respectively. **(B)** Structural comparison between an atomic model of AphB^*vp*^ based on cryogenic electron microscopy (map ID: EMD-60092) and AphB^*vc*^ (PDB ID: 3SZP). The tetrameric and monomeric structures of AphB^*vp*^ and AphB^*vc*^ are both highly similar. **(C)** Hypothesized resveratrol and ribavirin binding sites on tetrameric AphB^*vp*^. Molecular docking analysis was performed using Maestro with standard settings [100]. Note: This is based on studies on *V. cholerae* AphB [88,89]. Further study is needed before resveratrol and ribavirin can be used for AHPND prevention and treatment.

Since AphB^*vc*^ and AphB^*vp*^ share a high similarity in their amino acid sequences and structures (Fig 3A and 3B), ribavirin and resveratrol might also be useful in the prevention and treatment of AHPND. Molecular docking analysis suggests that these two compounds should both be able to bind with AphB^*vp*^ (Fig 3C), but this still needs to be confirmed experimentally. Finally, we should note that because AphB^*vp*^ becomes activated at low pH and induces the expression of the AHPND toxin genes, careful pH monitoring of the aquaculture pond water may also be required.

## Probiotics could be used to control AHPND by altering the QS system

Probiotic-mediated QS inhibition has been suggested as a promising, eco-friendly strategy to address antimicrobial resistance in aquaculture [90,91]. In other contexts, probiotics have already been shown to control pathogenetic bacteria by targeting their QS system. For instance, several strains of *Lactobacillus acidophilus* have exhibited anti-Quorum Sensing activity against *Clostridium difficile*, *Staphylococcus aureus*, and *E. coli* [92–94]. This was achieved either through the inhibition of AI-2 production or the downregulation of biofilm-related genes [95,96]. Another probiotic strain, *Bacillus velezensis* D-18, shows quorum quenching ability against a marine pathogen. In this case, *B. velezensis* D-18 acted by restraining bacterial growth and controlling the biofilm formation of the pathogenic vibrio *V. anguillarum* [97].

One of the proposed probiotics for AHPND control, *V. alginolyticus*, was evaluated as a means of enhancing shrimp resistance to this disease [24]. Two anti-QS compounds, Cyclo-(L-Leu-L-Pro) and Cyclo-(L-Phe-L-Pro), were identified in the *V. alginolyticus* extract, and they have both been confirmed to reduce the virulence of AHPND-causing *V. parahaemolyticus* [24] (Fig 2B). Specifically, these compounds are thought to interfere with or competitively inhibit the binding of AHPND-causative *V. parahaemolyticus* autoinducers (HAI-1, AI-2, and CAI-1) to their respective histidine kinase receptors (LuxN, LuxPQ, and CqsS). Such interference would be expected to disrupt QS signal transduction, leading to LuxO dephosphorylation and inactivation. Also, OpaR is upregulated with repression of AphA, leading to modulation of downstream QS-controlled phenotypes. These include a decrease in motility, biofilm formation, extracellular polymeric substance (EPS) production, capsular polysaccharide production, and virulence-associated functions, ultimately impairing colonization and PirAB^*vp*^ toxin delivery [24]. Other probiotic strains, such as *V*. *diabolicus* ILI [98], *B. subtilis* K3 [99] have also been shown to mitigate AHPND. Further investigation is warranted to determine whether these results are achieved by adjusting the QS system.

## Concluding remarks

In this review, we have summarized the current understanding of how Quorum Sensing is involved in AHPND pathogenesis. Understanding the QS system in *V. parahaemolyticus* not only provides insights into the environmental and cellular signals that triggers the pathogenesis of AHPND but also offers new and targeted opportunities for developing disease control strategies in aquaculture. These opportunities include identification of quorum quenching compounds from natural products and synthetic compounds, probiotics, and small molecule inhibitors for AphB^*vp*^. Further exploration of these mechanisms, especially the design of a suitable drug candidate, *in vitro* and *in vivo* validation of new QS inhibitors, toxicity testing, and the development of delivery methods in an aquaculture setting is recommended. Also, understanding how pond parameters such as pH, temperature, oxygen level, and salinity influence the QS system offers a complementary approach by providing a suitable environment that is less favorable for virulence and biofilm formation. All of these considerations are potentially important if we are to effectively translate these strategies to practical solutions to mitigate this major threat to shrimp aquaculture.
